# Supplementary figures and images for: A Gold Standard-Derived Modular Barcoding Approach to Cancer Transcriptomics
Source: Cancers (Basel). 2024 May 15;16(10):1886. doi: 10.3390/cancers16101886 (PMC11120226; doi:10.3390/cancers16101886)

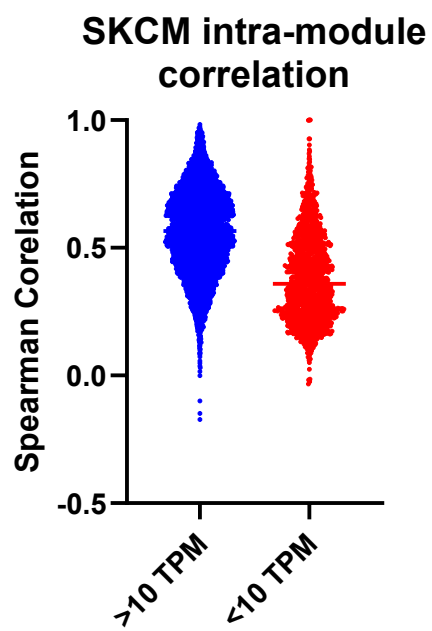

Supplement: Supplementary file 1 [file cancers-16-01886-s001.zip › New Modular Barcoding Supplemental Figure S1.pdf]

BLCA

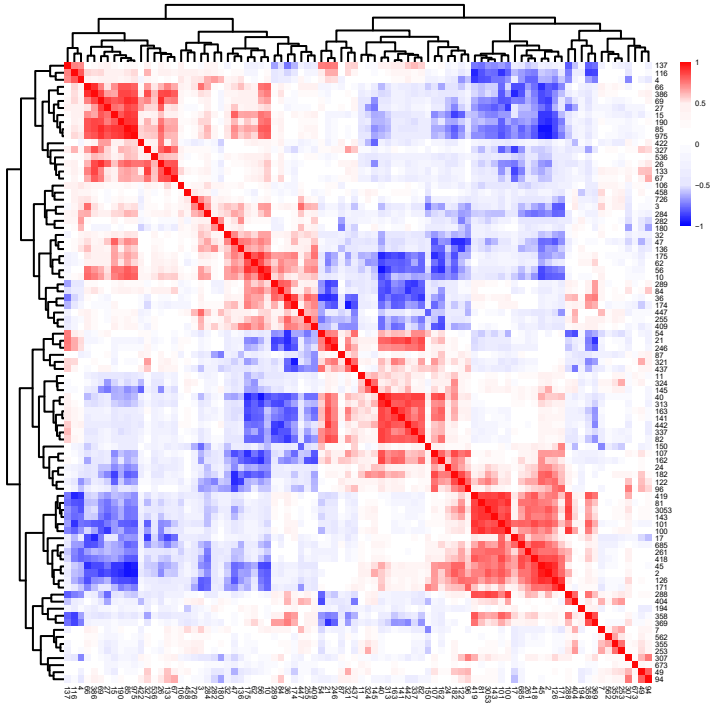

GBM

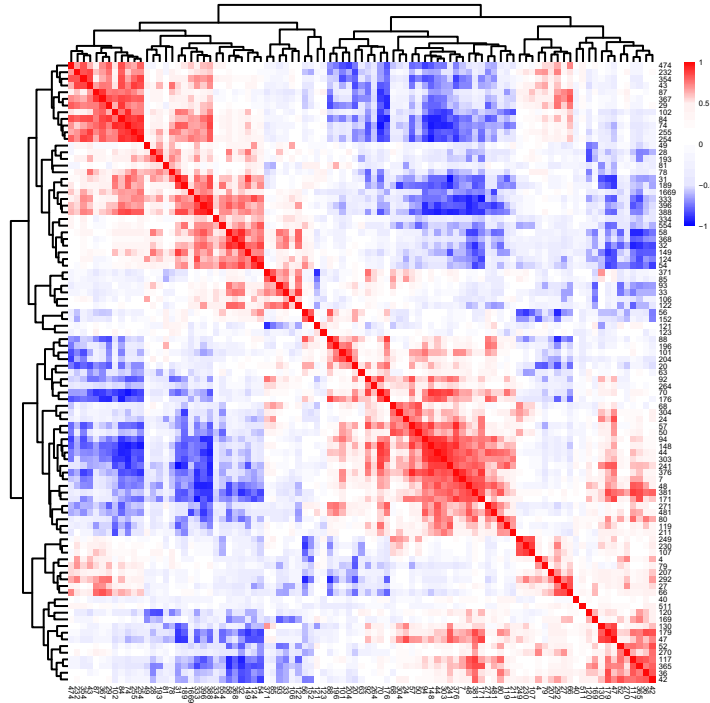

KIRC

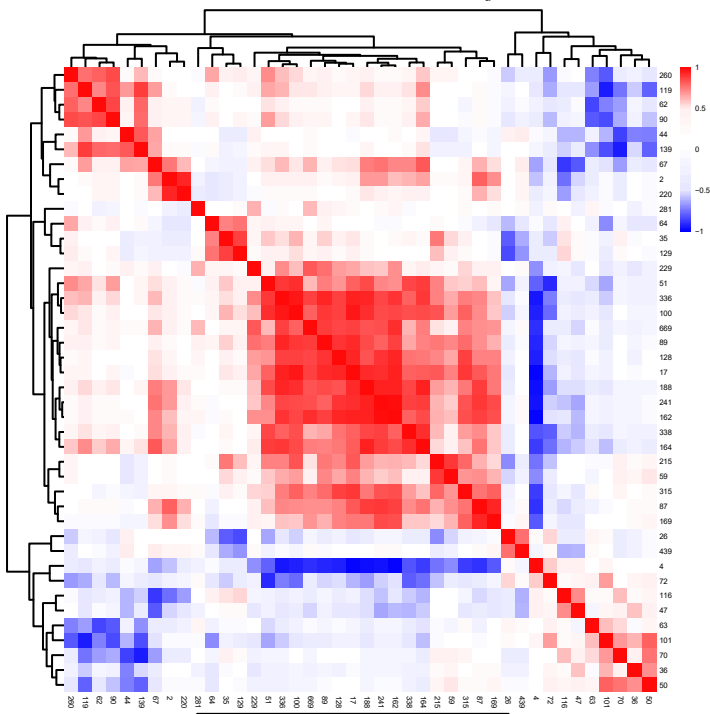

KIRP

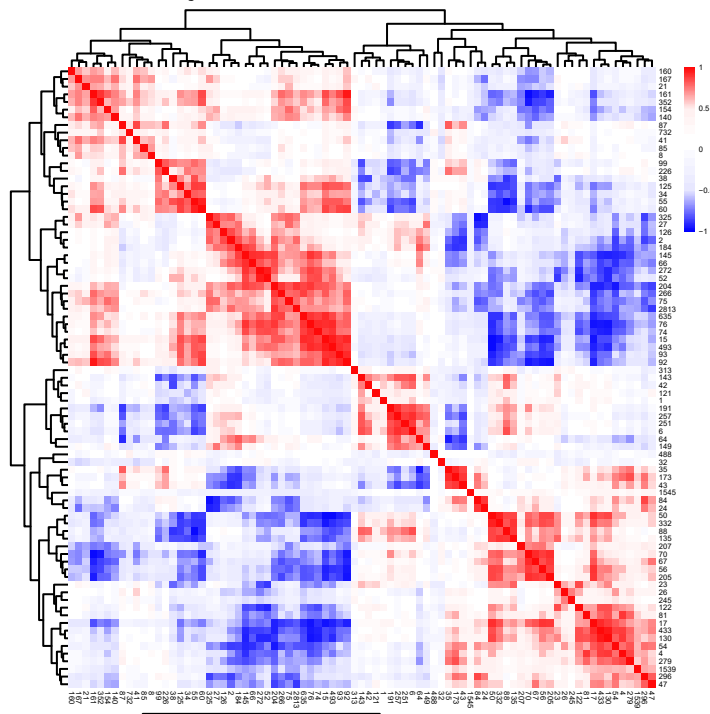

LGG

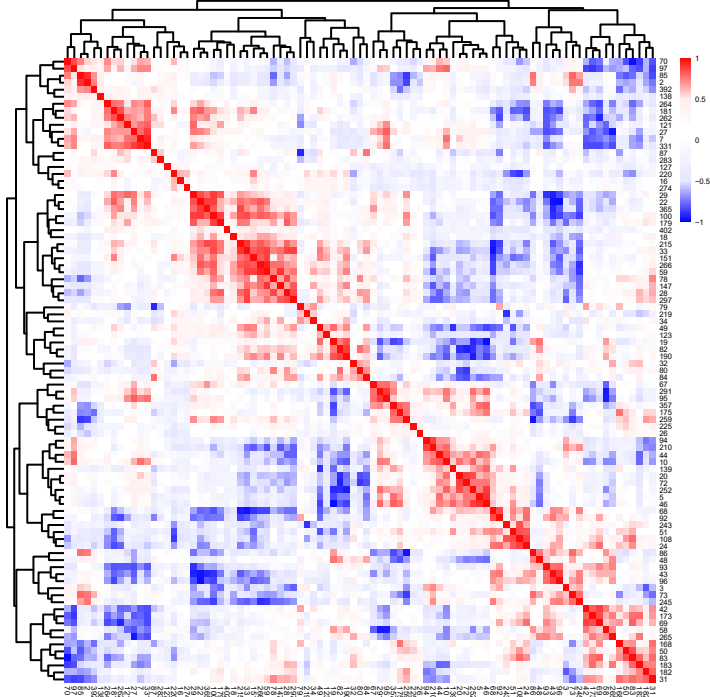

LHC

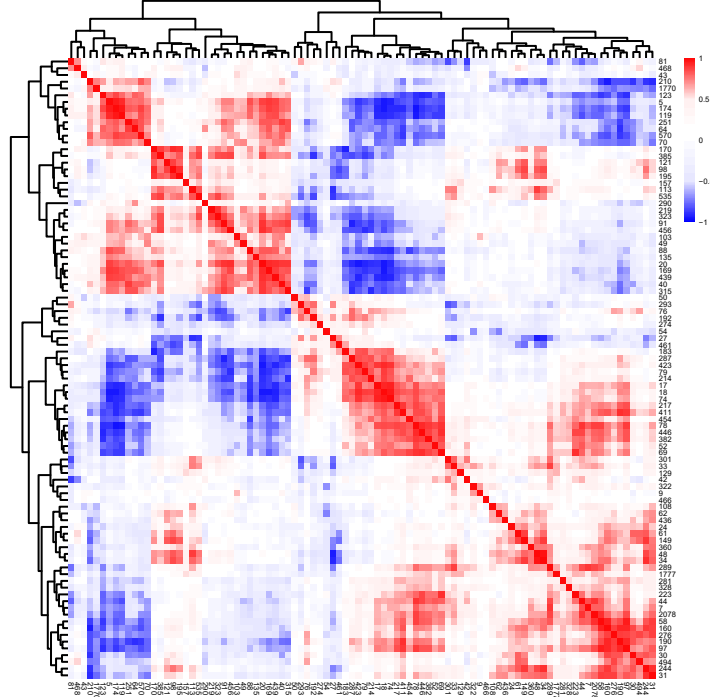

Supplement: Supplementary file 1 [file cancers-16-01886-s001.zip › New Modular Barcoding Supplemental Figure S2a.pdf]

TCGA

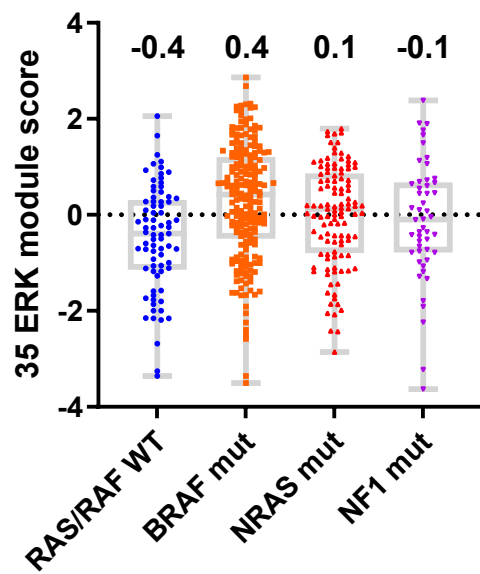

GSE22155

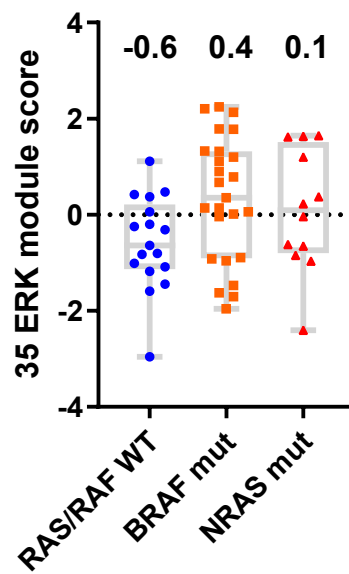

GSE15605

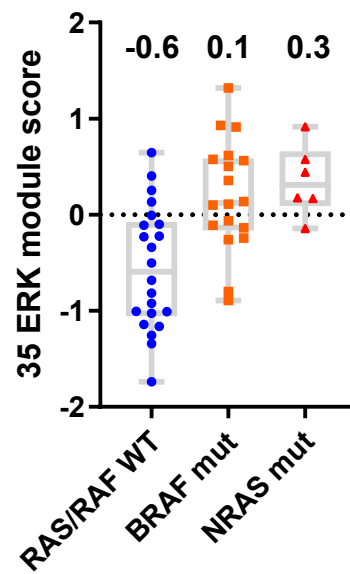

Supplement: Supplementary file 1 [file cancers-16-01886-s001.zip › New Modular Barcoding Supplemental Figure S3.pdf]

# A SKCM miRNAs

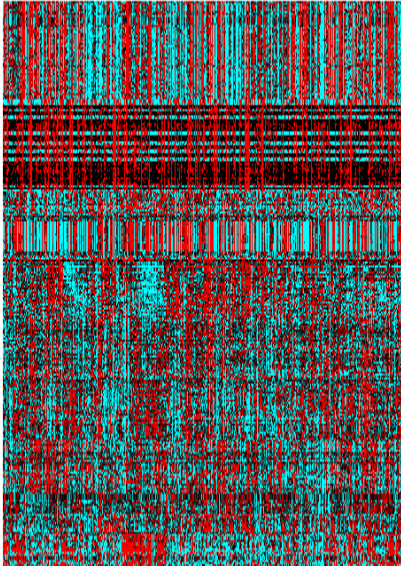

# B

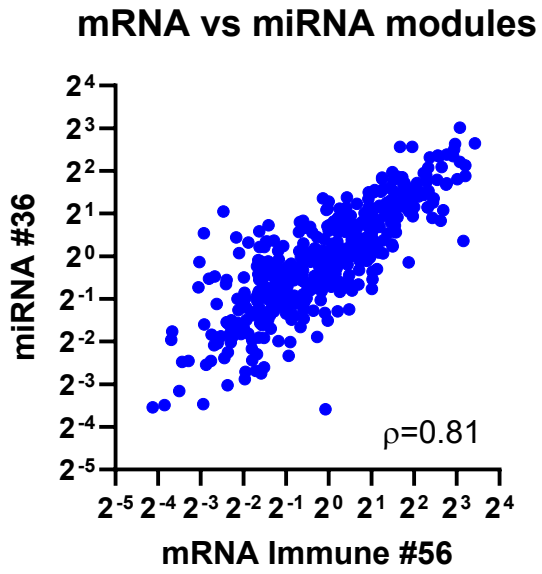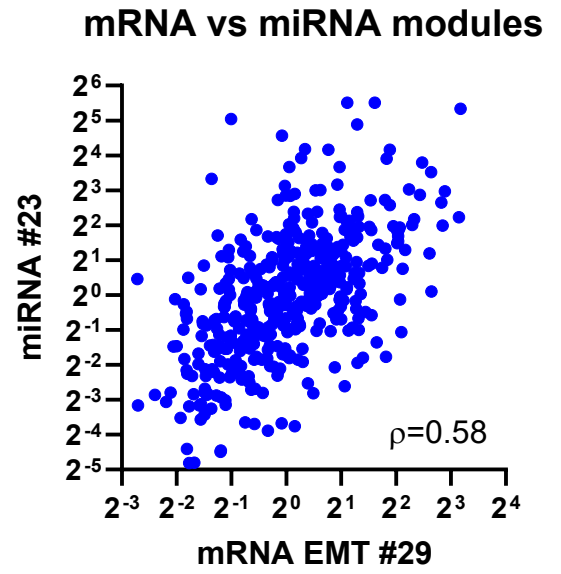

Supplement: Supplementary file 1 [file cancers-16-01886-s001.zip › New Modular Barcoding Supplemental Figure S4.pdf]

A

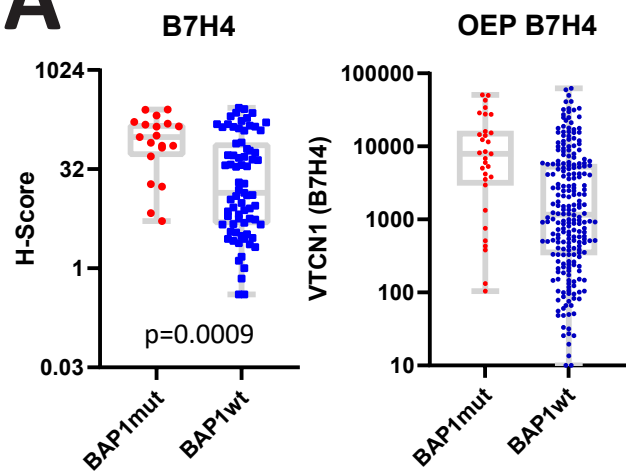

Supplement: Supplementary file 1 [file cancers-16-01886-s001.zip › New Modular Barcoding Supplemental Figure S5.pdf]

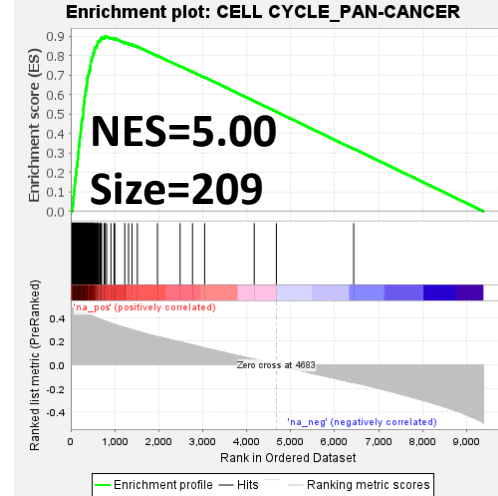

**c2**

**c5**

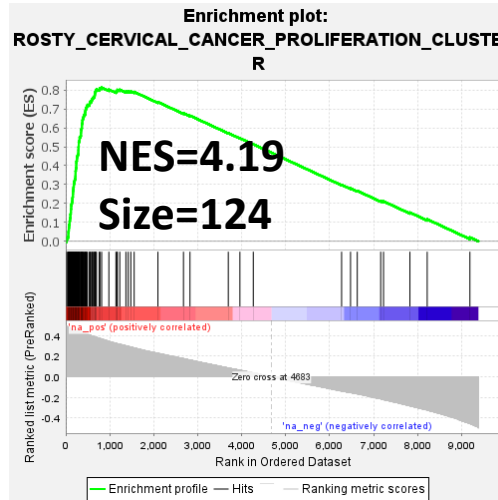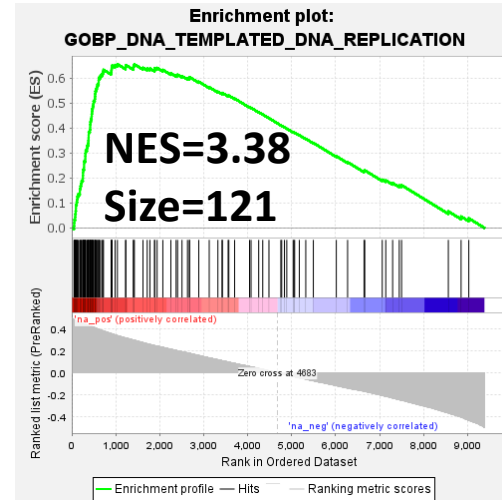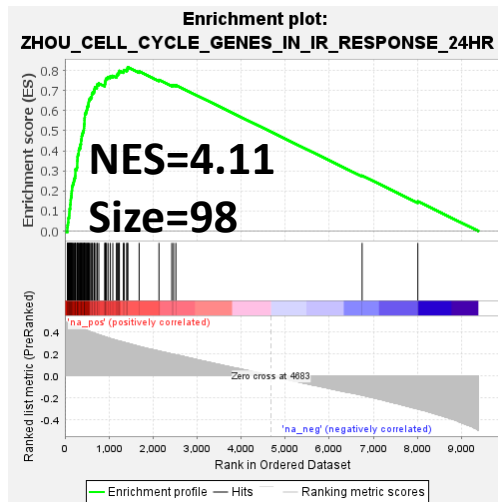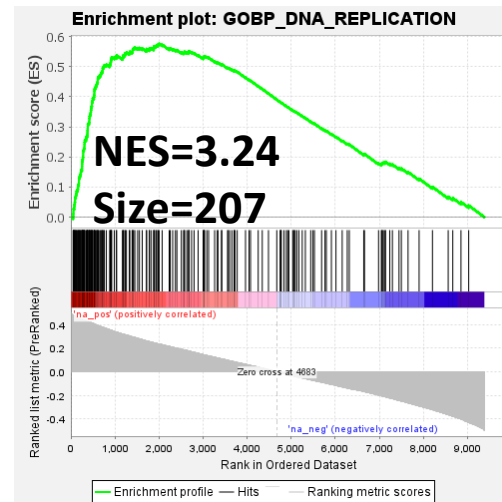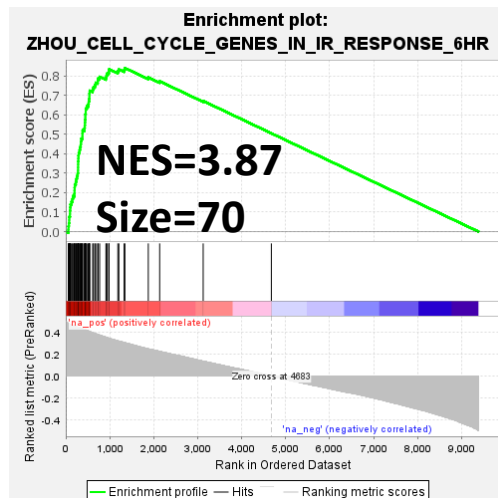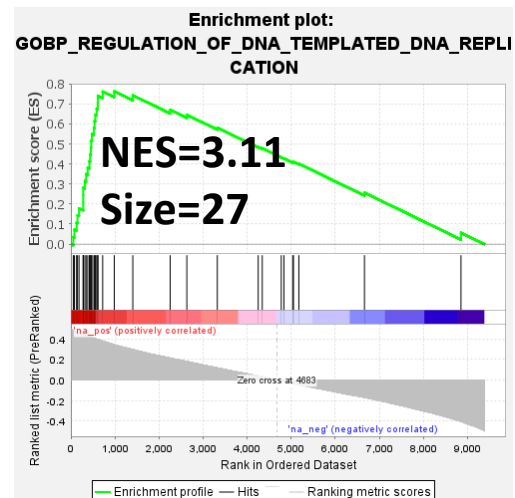

Supplement: Supplementary file 1 [file cancers-16-01886-s001.zip › New Modular Barcoding Supplemental Figure S6.pdf]
