# Supplementary material for: A Gold Standard-Derived Modular Barcoding Approach to Cancer Transcriptomics
Source: Cancers (Basel). 2024 May 15;16(10):1886. doi: 10.3390/cancers16101886 (PMC11120226; doi:10.3390/cancers16101886)
Supplement: Supplementary file 1 [file New Modular Barcoding Supplemental Figure S2b.pdf › New Modular Barcoding Supplemental Figure S2b.pdf]

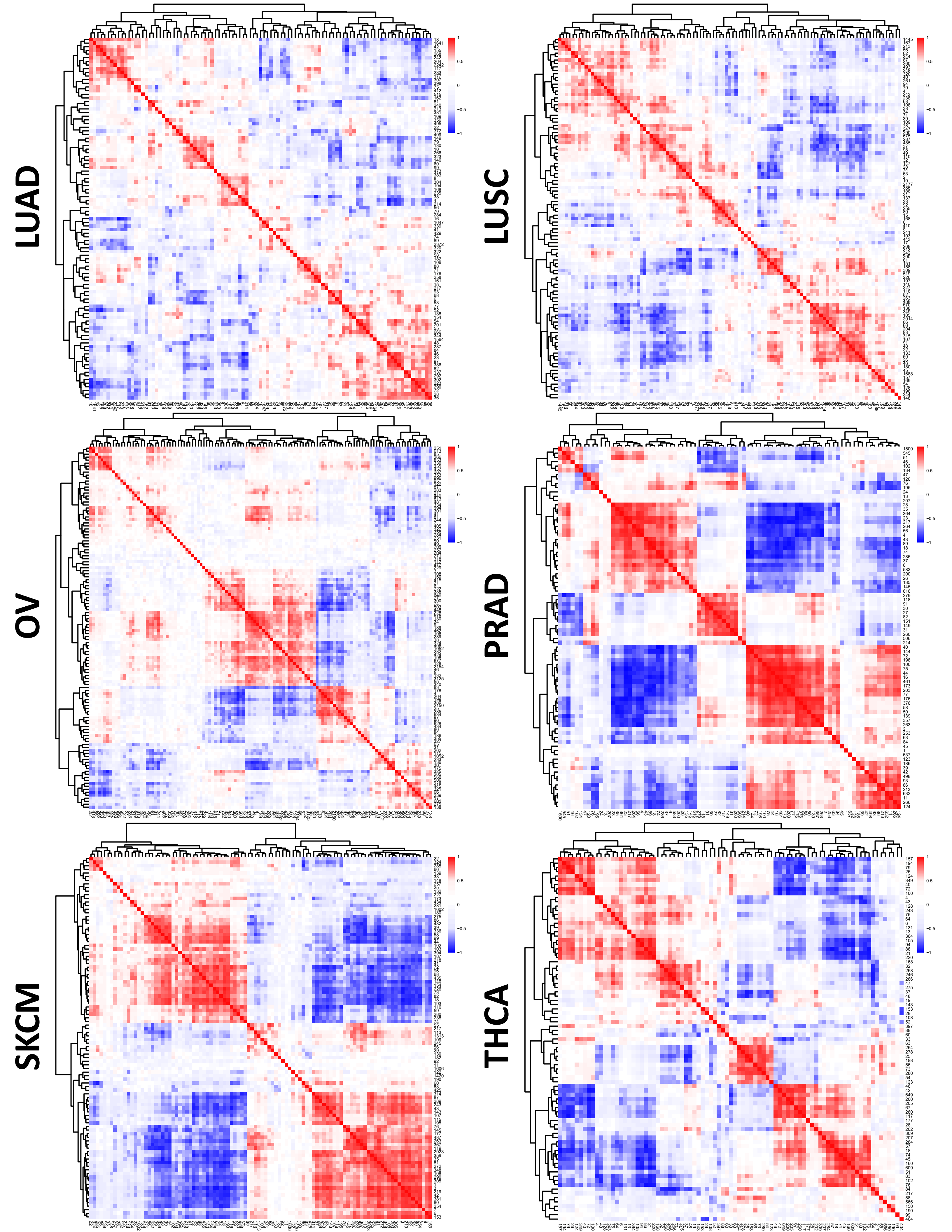
LUAD
LUSC
OV
PRAD
SKCM
THCA
